# Will mesenchymal stem cells be future directions for treating radiation-induced skin injury?

**DOI:** 10.1186/s13287-021-02261-5

**Published:** 2021-03-12

**Authors:** Zhuoqun Fang, Penghong Chen, Shijie Tang, Aizhen Chen, Chaoyu Zhang, Guohao Peng, Ming Li, Xiaosong Chen

**Affiliations:** Department of Plastic Surgery, Fujian Medical University Union Hospital, 29 Xinquan Road, Fuzhou, 350001 Fujian People’s Republic of China

**Keywords:** Mesenchymal stem cells, Radiation, Radiotherapy, Skin injury, Treatment

## Abstract

Radiation-induced skin injury (RISI) is one of the common serious side effects of radiotherapy (RT) for patients with malignant tumors. Mesenchymal stem cells (MSCs) are applied to RISI repair in some clinical cases series except some traditional options. Though direct replacement of damaged cells may be achieved through differentiation capacity of MSCs, more recent data indicate that various cytokines and chemokines secreted by MSCs are involved in synergetic therapy of RISI by anti-inflammatory, immunomodulation, antioxidant, revascularization, and anti-apoptotic activity. In this paper, we not only discussed different sources of MSCs on the treatment of RISI both in preclinical studies and clinical trials, but also summarized the applications and mechanisms of MSCs in other related regenerative fields.

## Introduction

Radiation-induced skin injury (RISI) is a common side effect of radiotherapy (RT) for malignant tumors or bone marrow transplant. Seventy percent of cancer patients especially with head and neck, skin, anogenital, and breast cancer would receive RT alone or as an adjuvant to surgeries at different stages and almost 95% patients with RT would occur with RISI [1, 2].

**Fig. 1:**
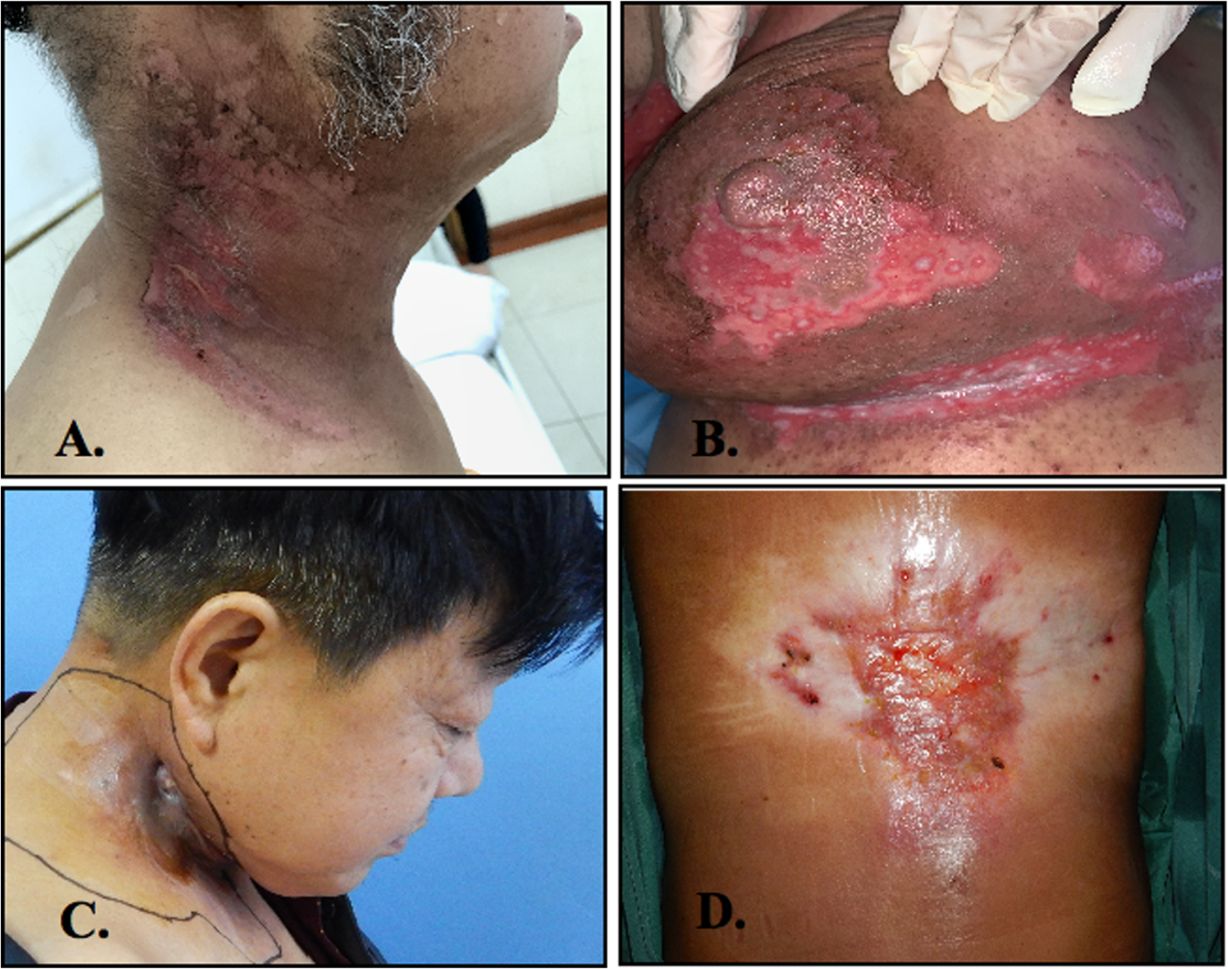
Four typical cases of radiation-induced skin injury. **a** Radiation-induced dermatitis appeared in a 61-year-old female patient who was diagnosed with esophageal cancer (*T3N0M0*) after the third RT. **b** Radiation-induced dermatitis appeared in a 59-year-old female patient suffering from mammary cancer (*T1N0M0*) after four times of RT. **c** Radiation-induced skin ulcer appeared in a 57-year-old male patient who suffered from esophageal cancer (*T4N0M0*) after 9 times of RT and conventional dressing change. **d** Radiation-induced skin ulcer appeared in a 44-year-old male patient with diagnosis of mycosis fungoides after 6 times of RT together with conventional dressing change

RISI is divided into acute RISI (occurring within hours to weeks after radiation) with clinical manifestations of skin erythema, blister, dermatitis, and necrotic ulcer and chronic RISI (occurrence from months to years post-radiation) with clinical syndromes of fibrosis, leathery skin, atrophy, and pigmentation [3]. These clinical symptoms mentioned above would not only reduce quality of life, but also interrupt tumor treatment progress (Fig. 1). Though traditional methods including topical steroids, creams, ointments, and hydrogel dressings have been applied to treat RISI, no criterion standard exists for the treatment of RISI [4–6].

In this paper, we discussed the applications and potential mechanisms of MSCs in RISI and summarized the applications of MSCs in other regenerative fields.

### The mechanism of RISI

**Fig. 2:**
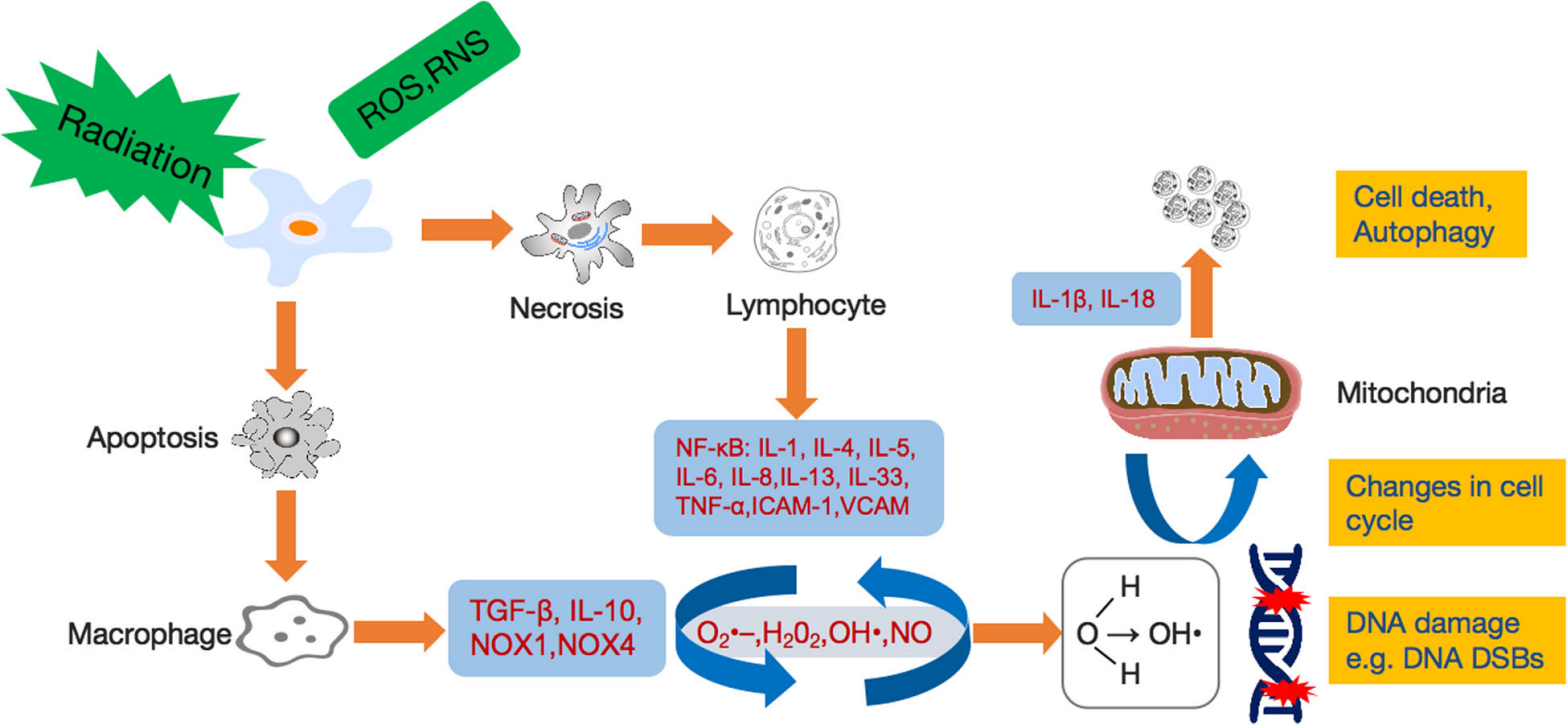
The molecular mechanisms of RISI

The mechanisms involved in RISI include oxidative stress and inflammation. The specific mechanisms of RISI are not fully understood but may be related to DNA damage, changes in cell cycle progression, and/or cell death [7, 8]. Chemical bond of DNA is particularly vulnerable to break while directly be exposed to radiation and the DNA double-strand breaks (DSBs) are the most serious type of DNA damage, from which γ-H2AX and MRN complex contains Mre11 are early indexes to reflect DNA damage and summarized in Fig. 2 [9–11].

#### Oxidative stress

Oxidative stress is the combination of cell damage and stress response signals that has been taken into account as one of the causes of acute and chronic radiation damage to the body. Reactive oxygen species (ROS) are additional products of aerobic metabolism varieties [10, 11]. Active oxygen-containing metabolites include superoxide anions (O_2_•–), hydrogen peroxide (H_2_O_2_), hydroxyl radicals (OH•), nitric oxide (NO), and its free radicals produced by its reaction with superoxide or oxygen that are known as reactive nitrogen species (RNS) [12–14]. Many reactions mediated by ROS protect cells from oxidative stress and then re-establish “redox homeostasis”. The increase of glutathione, an intracellular antioxidant, or the increase of protein/enzyme expression ability of scavenging ROS in cells is called oxidative stress reaction to maintain redox homeostasis in cells and tissues [15–17].

Some studies show that radiation damage to a single cell could trigger some signals and amplify the production of free radicals in irradiated cells that last for hours to days or even months [18, 19]. In addition, signals generated by irradiated cells could also be transferred to neighboring non-irradiated cells through bystander effects and activate REDOX metabolism [20]. Cytokines, miRNAs, vesicles, high mobility group box 1 (HMGB1), and free DNA fragments are those intracellular mediators by which chronic ROS/RNS released from irradiated cells, resulting in cell damage [21–23].

#### Inflammation

ROS/RNS could injure biological molecules such as nucleic acids, proteins, and lipids which would trigger a series of inflammatory reactions, result in apoptosis, and inhibit cell proliferation [24, 25]. Epithelial cells are extremely sensitive and susceptible to radiation as well as blood vessel and cutaneous appendages [26]. Free radicals generated from the interaction between radiation and water molecules may cause damage to biologically active ingredients. In the early stage of radiation injury, epithelial dysplasia, cell necrosis, nuclear pyknosis, and tissue fibrosis could be observed under the microscope [27, 28]. What is more, DNA DSBs caused by radiation would trigger changes in cell cycle progression, including the disturbing G1/S process and manifesting G1 cyclin inhibition. Some experiments showed that G1 inhibition was related to the survival state of p53 gene [29–31]. Upon cell exposure to radiation during DNA replication, it would extend the length of or lead to a block in S phase during DNA replication. Furthermore, ataxia telangiectasia mutated (ATM) and DNA-dependent protein kinase catalytic subunit (DNA-PKcs) are activated by DNA DSBs, followed by phosphorylation of checkpoint kinase 2 (CHK2) and lead to a block in G2/M phase [32–34]. Cell death events caused by radiation include apoptosis, necrosis, autophagy, mitotic catastrophe, and ferroptosis are related to the sensitivity of different cell types to radiation. Although the common mechanism may be related to damage of nuclear DNA, the specific mechanism involved in cell death still needs further research [35–37].

### Conventional treatment for RISI

Treatment details containing pros and cons of conventional methods for RISI are summarized in Table 1.

**Table 1:** Summary of the pros and cons of conventional treatments for RISI

Conventional treatments	Options	Pros	Cons	Reference
Systemic therapy	Antibiotics	Control and eliminate bacterial infection	Potential risk of antibiotic resistance occurrence	[38–40]
	Water-electrolyte balance	Supplement of loss of water-electrolyte by wounds	Cardiopulmonary load by excessive rehydration	[39, 41]
	Nutritional support	Reduce autologous protein degradation and enhance immunity	Burden to kidney and liver	[40, 42]
Local treatment	Topical corticosteroids	Anti-inflammation, immunosuppression and anti-proliferation	Decrease histamine and deplete mast cells on skin	[41, 43]
	Creams and ointments	Anti-inflammatory and promote macrophages recruitment	Less effective in managing patient-reported symptoms	[44, 45]
	Hydrogel dressings	Induce healing time, reduce pain and infection	Less frequent occurrence of severe skin reactions	[46, 47]
	Hyperbaric oxygen therapy	Increase the oxygen supply, inhibit inflammatory reaction, and reduce exudation	Arterial insufficiency, refractory osteomyelitis and bone necrosis	[48, 49]
	Superoxide dismutase	Antioxidant enzymes for scavenging free radicals	Not mentioned	[50, 51]
	Low-intensity laser	Reduction of edema and analgesia induction	Not mentioned	[50, 52]

### Systemic therapy

Systematic treatment is mainly based on different stages of RISI development, including antibiotics, water-electrolyte balance, and nutritional support. Experience has shown that infusion of fresh-frozen plasma (FFP), proteinase inhibitors, free-radical scavenger, and drugs of increasing the immunity could alleviate symptoms of RISI patients [38, 39].

### Local treatment

#### Topical corticosteroids

Corticosteroids are widely used to treat dermatitis induced by acute radiation [40]. Shukla et al. reported that the use of corticosteroids could prevent radiation-induced skin toxicity effectively and the incidence of wet desquamation was 13% in the corticosteroid group than 37% in control group [42]. Another clinical trial with 120 breast cancer patients enrolled showed that (mometasone furoate) MFF group significantly reduced radiation dermatitis and improved health-related quality of life [41].

#### Creams and ointments

Some non-steroidal anti-inflammatory creams and ointments have been applied to attempt to relieve the symptoms of RISI. It has been shown that topical application of triethylamine has a significant advantage in the prevention of radiation dermatitis by early recruitment of macrophages and stimulation of granulation tissue in comparison with the control group [43, 44].

#### Hydrogel dressings

Hydrogel is a form of highly cross-linked polymer that is polymerized by hydrophilic and hydrophobic groups and exhibits an outstanding characteristic of biocompatibility [45]. Censabella et al. indicated that patients with breast cancer received hydrogel dressings during radiotherapy period and significantly reduced the incidence of radiation-induced moist desquamation in comparison with the D-panthenol group [46].

#### Hyperbaric oxygen therapy (HBOT)

Hyperbaric oxygen therapy (HBOT) could be applied to form a hyperoxia environment in wound surface, keep the wound skin dry, and improve local microenvironment. The use of HBOT directly destroys the living environment of anaerobic bacteria, reduces the chance of bacterial infection, and accelerates the regression of redness and swelling [47, 48].

#### Superoxide dismutase (SOD)

SOD is a kind of antioxidant enzymes for scavenging free radicals in tissues by scavenging superoxide anion to reduce the adverse reactions [49]. Campan et al. reported that skin fibrosis induced by RT in breast cancer patients could be alleviated effectively with topical use of SOD [50].

#### Low-intensity laser

Main mechanisms of low-intensity laser for treating radiation dermatitis mainly are related to the expansion in local capillary, increase in vascular permeability, promotion of blood circulation, and proliferation of wound fibroblasts [51, 52].

### MSCs for applications in regenerative medicine

MSCs are multipotent stem cells that can differentiate into a variety of cell types to maintain tissue integrity and intracellular homeostasis [53]. On-going clinical applications involved adipose-derived mesenchymal stem cells (ADMSCs), stromal vascular fractions (SVFs), and platelet-rich plasma (PRP) have raised in regenerative fields enrolling wound healing, scar management, breast augmentation, and soft tissue defects reconstruction [54, 55]. MSC actions through anti-inflammatory and immune-modulatory capabilities by secreting large number of anti-inflammatory cytokines, cell growth factors, antibacterial peptides, and proteins such as IL-17, indoleamine 2,3-dioxygenase (IDO), and other functional molecules [56, 57]. The application of MSCs to patients after the SARS-CoV-2 infection could alleviate the over-activation of the immune system, improve the lung microenvironment, and reduce the risk of cytokine storm syndrome (CSS) and acute respiratory distress syndrome (ARDS) of the body [58–60]. Animal experiments and clinical applications using MSCs in the treatment of RISI were summarized in Tables 2 and 3.

**Table 2:** Summary of MSC source, injury type, and main findings from the treatment of RISI using MSCs in preclinical studies

Publication	Animal model	MSC source	Injury type	Main findings	Reference
Rong et al. 2019	Sprague-Dawley rat	Human fetal skin-derived stem cell	Radiation-induced skin injury	Enhanced radiation dermatitis angiogenesis	[1]
Kakabadze et al. 2019	Lewis inbred rats	Rat bone marrow	Nonhealing wounds of radiation	Closing the burn wound increased the rate of healing	[4]
Myung et al. 2019	SKH-1 hairless mice	Umbilical cord blood-derived	Combined radiation and wound injury	UCB-MSCs+PRP improve regeneration efficacy by enhancing angiogenesis	[7]
Wu et al. 2018	Nude mice	Adipose-derived stem	Cutaneous punch wounds with radiation exposure	Revitalized irradiated tissues and accelerated wound healing	[10]
Sun et al. 2018	Sprague-Dawley rat	WJ-MSCs	Radiation-induced skin injury	Accelerated wound closure and enhanced the wound healing quality	[16]
Liu et al. 2018	SD rats	Human umbilical cord	Irradiation-induced skin ulcers	Promoted neovascularization and reepithelization, and improved healing of irradiation-induced skin ulcers	[13]
Lee et al. 2017	Male C57BL/6 mice	Human umbilical cord blood-derived	Combined radiation wound	Enhanced wound healing and angiogenesis in the wound site	[15]
Riccobono et al. 2016	Minipigs	Adipose tissue-derived stromal/stem cells	Cutaneous radiation syndrome	Have an effect on both muscle inflammation and regeneration	[20]
Jin et al. 2016	Sprague-Dawley rat	Rat MSCs+PDGF	Radiation-induced skin ulceration	Radiation-induced skin ulceration	[22]
Rodriguez-Menocal et al. 2015	C57BL/6 mice	Whole bone marrow, whole bone marrow cultured cells, MSCs	Radiation-induced delayed wound	Mixed bone marrow cell preparations may be superior to a more purified stem cell formulation in stimulating wound healing	[23]
Horton et al. 2013	C3H/HeN mice	Syngeneic or allogeneic BMSC	IR-induced fibrosis	Alter the progression of radiation-induced fibrosis by altering macrophage phenotype and suppressing local inflammation	[61]
Huang et al. 2013	Sprague-Dawley rat	Adipose-derived stem cells	Acute radiation ulcers	Contributed to vascularization by acting as angiogenesis-promoting cells	[26]
Forcheron et al. 2012	Minipigs	Autologous adipose MSCs	Cutaneous radiation syndrome	Attracted numerous immune cells and accumulated at the dermis/subcutis barrier	[27]
Agay et al. 2010	Minipig	Autologous bone marrow	Cutaneous radiation syndrome	Led to local accumulation of lymphocytes and improved vascularization	[30]
Riccobono et al. 2012	Minipig	Adipocyte-derived stem cells	Cutaneous radiation syndrome	Improved wound healing	[32]
Horton et al. 2013	Female C3H/HEN mice	Mouse bone marrow	Cutaneous radiation syndrome	Reduced inflammation and fibrosis	[33]
Zheng et al. 2015	Sprague-Dawley rat	Rat bone marrow	Radiation-induced skin injury	Reduced inflammation, decreased expression of PGE2 and TGF-β1	[38]
Xia et al. 2014	Sprague-Dawley rat	Rat bone marrow MSCs expressing human VEGF and beta-defensin-3	Radiation and excisional injury	Improved skin appendage regeneration and collagen deposition; shortened wound healing duration	[45]
Xie et al. 2013	C3Hf/Kam female and C57Bl/6 male gnotobiotic mice	Murine bone	Radiation-induced wound	Haptized collagen strips to reduce radiation wound-healing deficits	[54]
Kotenko et al. 2012	Male Wistar rats	Rats bone marrow	Radiation skin damage	Normalize intercellular interaction and influence the processes of proliferation and differentiation of skin cells	[8]
Yan et al. 2011	Minipigs	MSCs expressing hPDGF loaded onto acellular amniotic membrane	Radiation and excisional injury	Improved granulation, re-epithelialization and angiogenesis	[62]
Ebrahimian et al. 2009	C57Bl/6 male mice	Homogenous ADSCs	Skin punched wounds and then irradiated	Promoted dermal wound healing and enhanced wound closure	[63]
Hao et al. 2008	Sprague-Dawley rat	Rat bone marrow MSCs expressing human PDGF and beta-defensin-2	Radiation and excisional injury	Improved granulation tissue formation and reduced bacterial load	[64]
Francois et al. 2007; Mouiseddine et al. 2007	NOD/SCID mice	Human bone marrow	Radiation dermatitis	Improved wound healing	[65]

**Table 3:** Summary of MSC source, injury type, and main findings from the treatment of RISI using MSCs in clinical applications

Publication	MSC source	Combined treatment	Injury type	Main findings	Reference
Portas et al. 2016	Allogenic cadaveric bone marrow	Surgical methods, hyperbaric oxygen treatment	Radiation-induced chronic skin lesions	Ulcer dimensions were reduced and remission of signs and symptoms	[2]
Guo et al. 2014	Allogeneic bone marrow	HLA-mismatched peripheral blood stem cell transplantation	Radioactive skin ulceration	Suggesting a potential benefit of MSCs in radiation treatments	[5]
Kotenko et al. 2012	Human bone marrow	Surgery and traditional conservative therapy	Severe local radiation ulcers	Rapid growth of granulation tissue and marginal epithelization and reduction in the ulcer	[8]
Bey et al. 2010	Autologous bone marrow	Skin autograft	Severe radiation burn and radiation dermatitis	Modulating radiation inflammatory processes	[12]
Benderitter et al. 2010	Autologous bone marrow	Plastic surgery or skin graft	Severe radiological burn	Being driven by the quality and the rapidity of the wound healing	[14]
Akita et al. 2010	Autologous adipose tissue	Temporal artificial dermis impregnated+BFGF	Chronic radiation injuries	Wound was healed and no sign of recurrence appeared	[17]
Lataillade et al. 2007	Autologous bone marrow	Surgery	Radiation burn	Open new prospects in radiotherapy complications	[19]

### Adipose-derived mesenchymal stem cells (ADMSCs)

ADMSC is one of the sources of stem cells which could secrete a variety of cytokines with multiple differentiation potential and immune exemption features. ADMSCs are extracted from adipose tissue and have biological characteristics of convenient obtain access [66–68]. Pietro et al. identified the phenotype of MSCs and elaborated that transcription factors (including OCT4, SOX2, NANOG, NEUROD1, PAX6, and SOX3) were involved in self-renewal capacity and multi-lineage differentiation potential. They verified the safety and efficacy of human ADMSCs localize in SVFs in breast reconstruction and assessed the volumetric persistence of breast augmentation by fat graft enhanced with ADMSCs in a 5-year follow-up retrospective case series [8, 69–73]. Besides, classic nanofat methods were innovatively modified by Pietro et al. and presented better outcomes of SVFs for the treatment of scars [62, 74–76]. They also proposed the maintenance and survival of SVF-enhanced autologous fat grafts were promoted by mixing with PRP and showed better outcomes of scars on the face compared with traditional surgical methods [63–65, 77]. Maria et al. combined PRP/insulin by improving chondrogenic and osteogenic differentiation of ADMSCs in a 3D collagen scaffold for treating osteochondral defects [78, 79].

Results of current applications with MSCs especially with ADMSCs indicated ADMSCs being new, alternative while bright approaches for treating those who suffer from the COVID-19 pandemic caused by coronavirus 2 (SARS-CoV-2) because of its easy access from autologous abdominal subcutaneous fat with low immunogenicity and little ethical disputes [80–82].

### Bone marrow-derived mesenchymal stem cells (BMMSCs)

BMMSCs own the ability to differentiate into osteoblasts, chondroblasts, tendon cells, endothelial cells, glial cells, and hepatocyte. In view of the unique clinical and biological characteristics such as secretion of various hematopoietic growth factors, reconstruction of hematopoietic microenvironment, low immunogenicity together with vulnerable to transfection, and expression of foreign gene products, BMMSCs could partly make up for the deficiency of traditional treatment [83, 84]. BMMSCs still maintain its multi-directional differentiation potential and induce amplification in a destroyed tissue environment to participate in tissue repair or regeneration process [70, 85].

### Umbilical cord mesenchymal stem cells (UCMSCs)

In recent years, UCMSCs have attracted more attention because of the stronger ability of proliferation and differentiation compared with BMMSCs. Apart from anti-inflammatory properties, UCMSCs play numerous roles in promoting systemic and local tissue repair, enhancing the ability of autophagy and self-repair together with increasing oxidative stress resistance in order to promote angiogenesis [86, 87]. Fang et al. studied that UCMSC-derived exosomes could reduce the myogenesis fibroblast accumulation and scar formation in a mouse wound defect model [88]. Wang et al. reported that human UCMSCs present positive treatment effects on acute radiation enteritis in rats [89].

## Discussions

Various reasons include RT, occupational exposure, nuclear leakage accident, or/and nuclear war would lead to RISI that characterized by potential progressiveness and difficult to heal [90, 91]. Stewart et al. indicated that radiation-induced changes of signal molecule levels and formation of oxygen free radicals would cause DNA single-strand breaks, resulting in incomplete repair, premature senility, and accelerated differentiation [92].

**Fig. 3:**
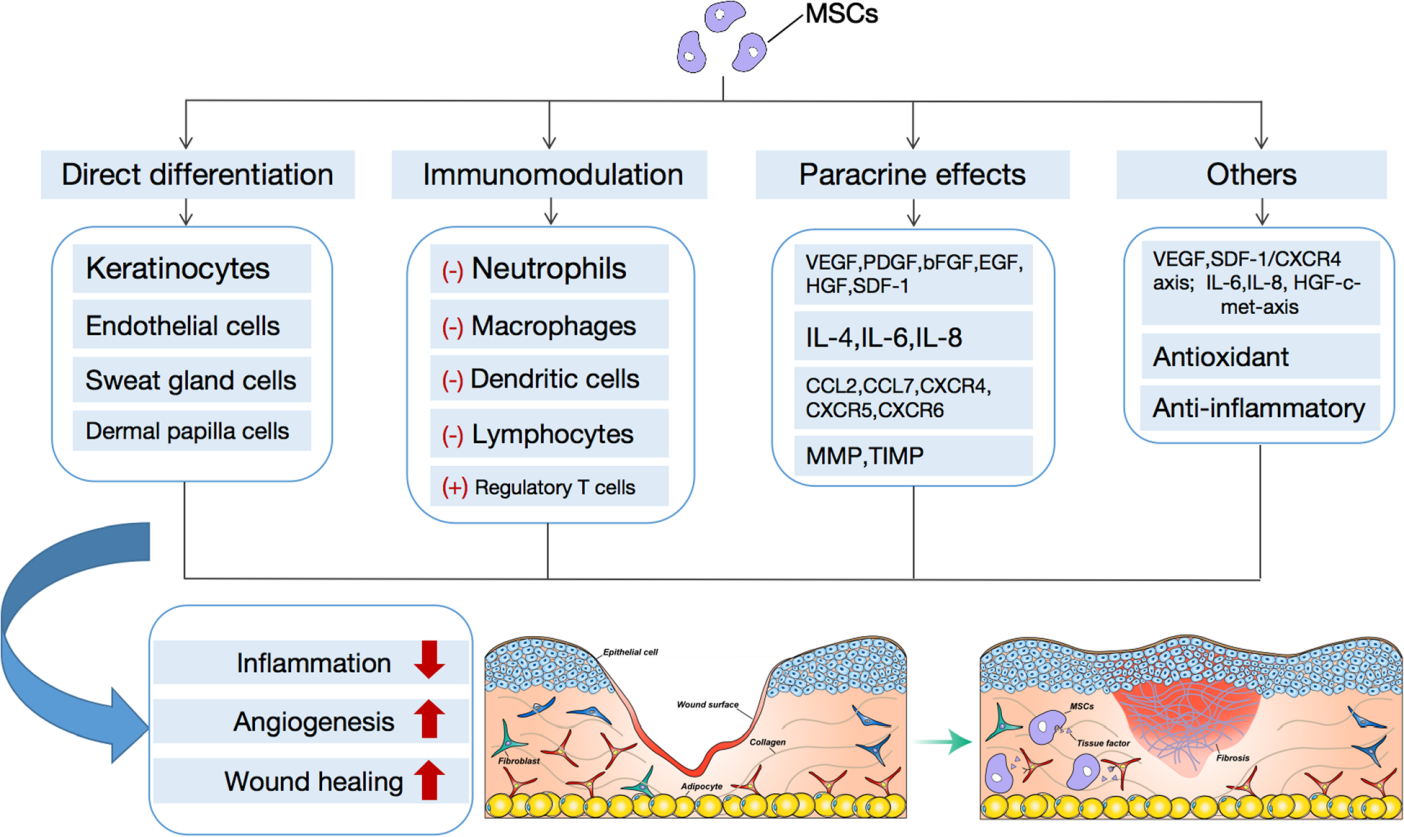
The mechanisms of MSCs in the field of regenerative medicine (e.g., in wound repair)

The mechanisms of MSCs involved in wound progression are mainly through direct differentiation, immunomodulation, paracrine actions, and other related mechanisms such as recruitment of endogenous stem/progenitor cells, antibacterial and antioxidant effects with the intent of improving the wound microenvironment, shortening the inflammatory phase, and accelerating the proliferation phase to regulate matrix deposition and collagen remodeling, so as to arrive the accelerated healing and less scar formation [41, 54, 93] (Fig. 3). The inflammatory cytokines (e.g., anti-inflammatory cytokines like interleukin-10 (IL-10) and interleukin-4 (IL-4) and inflammatory factors such as tumor necrosis factor α (TNF-α) and interferon β (IFN-β)) and immune cells of wounds could activate MSCs that associated with proliferation and secretion of different immune cell subsets. Besides, MSCs promote the transformation of macrophages from M1-type to M2-type to accelerate the process of wound repair [94–96].

MSCs can be differentiated into keratinocytes, endothelial cells, sweat gland cells, or dermal papilla cells to participate in wound repair after direct induction in vitro. ADMSCs were obtained from the adipose tissues and are characterized by abundant sources and easy access compared to other sources of MSCs such as BMMSCs and UCMSCs. Altman et al. indicated that ADMSCs with fluorescent protein marked to dermal scaffolds could detect the expression of heat shock protein 47 (HSP-47) through fibroblast differentiation [97]. Ebrahimian et al. detected the expression of keratin 5 (K5) and keratin 14 (K14) which are signs of keratinocytes in epidermis after injection of ADMSCs into the back muscles of mice and indicated the differentiation potential of ADMSCs to epidermal keratinocytes [63]. Because of their lower immunogenicity, with no or low expression of major histocompatibility complex (MHC), host immune reaction is rarely caused after transplantation of ADMSCs [60, 91].

In recent years, BMMSCs have been one of the research hotspots for regenerative medicine with the following biological characteristics: (1) BMMSCs were able to grow adhering to the wall of the culture dishes; (2) the cell surface phenotype were CD3, CD4, CD8, CD14, CD19, CD34, CD45, and HLA-DR negative and CD29, CD44, CD71, CD73, CD90, CD105, and CD124 positive; (3) medium levels of positive MHC I expression and no expression of MHC II; (4) no expression of costimulatory molecules like B7-1, CD40, and CD86 [60, 92–94, 98, 99]. Silva et al. reported that BMMSCs could differentiate into vascular endothelial cells in vivo microenvironment and result in re-epithelialization of the wounded tissues [100]. Recent research has shown that MSCs participate in wound repair through paracrine growth factors, cytokines, and chemokines which have been described before. Some researchers reported that BMMSCs could be induced in radioactive environments that lead to further amplification to participate in tissue repair and regeneration process, resulting in achieving the purpose of MSCs transplantation in the treatment of radiation injury [101–103]. HGF and PGE2 that secreted by MSCs could, on the one hand, regulate the balance of TGF-β1 and TGF-β2 on the wound surface and, on the other hand, inhibit the EMT process to inhibit the formation of myofibroblasts and lead to less scar outcomes.

Though BMMSCs have been widely studied in recent years, obtaining BMMSCs would bring risks to patients because of the invasive operation which might restrict the application of BMMSCs [104, 105]. Umbilical cord blood contains a large number of MSCs with multi-lineage differentiation potential and homing properties. UCMSCs could be derived from a wide range of sources with easier access and lower immunogenicity compared with other sources of MSCs [106, 107]. Human UCMSCs promote neovascularization and reepithelization to enhance the healing of radiation-induced skin ulcer might be activated by PI3K/Akt signaling pathway [108]. In addition, the result in the study indicated the regulatory effect UCMSCs to Flt-3L and TGF-β1. What is more, VEGF/SDF-1-CXCR4, inflammatory cytokines (e.g., IL-6 and IL-8), and growth factor receptor axis (e.g., HGF-c-met and PDGF axis) are involved in the recruitment of endogenous stem/progenitor cells of MSCs to promote tissue repair [83, 109–111].

## Conclusion

Many studies have confirmed the effects of MSCs on tissue repair as an acellular therapy in recent years. This review focused on the latest application of MSCs from various sources as a promising agent for RISI both in animal models and clinical trials and discussed related mechanism. The specific mechanism of MSCs on tissue repair and regenerative medicine need to be further studied in the future.

## Data Availability

Not applicable.

## Abbreviations

RISI: Radiation-induced skin injury
RT: Radiotherapy
MSCs: Mesenchymal stem cells
ROS: Reactive oxygen species
NO: Nitric oxide
RNS: Reactive nitrogen species
HMGB1: High mobility group box 1
ATM: Ataxia telangiectasia mutated
DNA-PKcs: DNA-dependent protein kinase catalytic subunit
FFP: Fresh-frozen plasma
HBOT: Hyperbaric oxygen therapy
CHK2: Checkpoint kinase 2
SVFs: Stromal vascular fractions
PRP: Platelet-rich plasma
SOD: Superoxide dismutase
VEGF: Vascular endothelial growth factor
IDO: Indoleamine 2,3-dioxygenase
PDGF: Platelet-derived growth factor
CSS: Cytokine storm syndrome
ARDS: Acute respiratory distress syndrome
ADMSCs: Adipose-derived mesenchymal stem cells
BMMSCs: Bone marrow-derived mesenchymal stem cells
UCMSCs: Umbilical cord mesenchymal stem cells
SARS-CoV-2: Coronavirus 2
IL-10: Interleukin-10
IL-4: Interleukin-4
TNF-α: Tumor necrosis factor α
IFN-β: Interferon β
HSP-47: Heat shock protein 47
K5: Keratin 5
K14: K14 keratin
MHC: Major histocompatibility complex
